# Changes of lysosomes in the earliest stages of the development of atherosclerosis

**DOI:** 10.1111/jcmm.12042

**Published:** 2013-03-14

**Authors:** Yuri V Bobryshev, Tatyana A Shchelkunova, Ivan A Morozov, Petr M Rubtsov, Igor A Sobenin, Alexander N Orekhov, Alexander N Smirnov

**Affiliations:** a Faculty of Medicine, School of Medical Sciences, University of New South WalesKensington, NSW, Australia; b School of Medicine, University of Western SydneyCampbelltown, NSW, Australia; c Institute for Atherosclerosis Research, Skolkovo Innovation CenterMoscow, Russia; d Biological Faculty, Lomonosov Moscow State UniversityMoscow, Russia; e Engelhardt Institute of Molecular Biology, Russian Academy of SciencesMoscow, Russia; f Russian Cardiology Research and Production ComplexMoscow, Russia; g Institute of General Pathology and Pathophysiology, Russian Academy of Medical SciencesMoscow, Russia

**Keywords:** atherosclerosis, initial lesion, fatty streak, aorta, intima, lysosomes, CD68

## Abstract

One of hypotheses of atherosclerosis is based on a presumption that the zones prone to the development of atherosclerosis contain lysosomes which are characterized by enzyme deficiency and thus, are unable to dispose of lipoproteins. The present study was undertaken to investigate the characteristics and changes of lysosomes in the earliest stages of the development of atherosclerosis. Electron microscopic immunocytochemistry revealed that there were certain changes in the distribution of CD68 antigen in lysosomes along the ‘normal intima-initial lesion-fatty streak’ sequence. There were no significant changes found in the key mRNAs encoding for the components of endosome/lysosome compartment in initial atherosclerotic lesions, but in fatty streaks, the contents of EEA1 and Rab5a mRNAs were found to be diminished while the contents of CD68 and p62 mRNAs were increased, compared with the intact tissue. The study reinforces a view that changes occurring in lysosomes play a role in atherogenesis from the very earlier stages of the disease.

## Introduction

Atherosclerotic lesions form as early as during childhood and continue to develop and transform throughout an individual's life 1. Despite a detailed understanding of the mechanisms that initiate the disease being crucial to our ability to find a preventive therapy, the characteristics and peculiarities of the earliest lesion type that precedes the formation of a fatty streak are poorly investigated 2, 3. The guidelines of the American Heart Association define the initial atherosclerotic lesion as a Type I lesion 2, 3. In contrast to a fatty streak (Type II lesion) that contains a focal aggregation of lipid-laden (foam) cells, in which several layers of foam cells can be distinguished, a Type I lesion contains only a small number of individually located foam cells which are not yet aggregated 2, 3. While fatty streaks can be easily detected macroscopically, the detection and identification of Type I lesions require the use of microscopic examination and this circumstance, in addition to other technical difficulties of the acquisition of samples of human early atherosclerotic lesions, explains why the information about the initial lesion type is scanty 2, 3.

The information gained from the available reports on the earliest alterations of the human arterial intima as well as the findings obtained from experimental models of atherosclerosis facilitated offering several hypothesis of atherosclerosis (Reviewed elsewhere 1, 4–10). One of the hypotheses, proposed by Christian de Duve 11–13, is based on a presumption that the zones prone to the development of atherosclerosis contain lysosomes which are characterized by enzyme deficiency and thus, are unable to dispose of lipoproteins. According to this hypothesis, a lysosomal deficiency or lysosomal defects lead to the accumulation of lipids within lysosomes and, eventually, to the formation of foam cells which are the earliest morphological hallmark of the development of an atherosclerotic lesion 11–14. De Duve has defined foam cell formation in atherosclerosis as a variant of lysosomal storage disease 11–13. De Duve's hypothesis was strengthened by the observations that cholesteryl esters, which are the major form of cholesterol in atherosclerotic lesions, cannot be cleared from lysosomes unless they are first hydrolysed to cholesterol and fatty acid 15, 16. In agreement with this, morphological studies have demonstrated that the cytoplasm of foam cells is filled with membrane-surrounded lipid inclusions and ‘lipid droplets’ that are not surrounded by membranes 17, 18. Classic lysosomal storage diseases are known to result from mutations in key digestive enzymes 19, but the question whether lysosomal mutations might be an essential attribute of atherosclerosis has not received so far much attention 20. Nevertheless, it is thought that it is extremely unlikely that mutations could be virtually in the entire population 16, 17. It is more likely that the failure of lysosomes in atherosclerosis may be a result of altered expression of genes relevant to lysosomes and other elements of the vacuolar-lysosomal system 16, 17. The question whether there might be some peculiarities in the structure and content of lysosomes in the very early stage of atherosclerosis has not yet been addressed.

In this study, analysing urgent autopsy tissue specimens of the human aorta, we focused on the examination of lysosomes in the very early stages of atherosclerotic alteration of the intima. Specifically, in this study we compared: (i) the properties of lysosomes in the initial lesion (Type I lesion) with those of the normal intima; (ii) the properties of lysosomes in fatty streaks (Type II lesion) with those of the normal intima and (iii) the properties of lysosomes in the initial lesion (Type I lesion) with those of fatty streak (Type II lesion). We investigated: (i) the structural peculiarities of lysosomes and (ii) the peculiarities of the system of inner translocation and storage of molecules that represent key markers of endosomes and lysosomes 21, 22. Because this system could be described not only by the level of interactions between protein components in these compartments but also by the level of expression of the corresponding genes 21, 22, in this study a number of the relevant genes were studied.

## Materials and methods

### Sample collection and initial processing

The material was collected in accordance with the ethical guidelines outlined in the Helsinki Declaration and the Medical Research Council's statement on responsibility in investigation on human volunteers. The study was approved by the ethics committees of the Institute for Atherosclerosis Research and the Russian Cardiology Research and Production Complex, Moscow.

For this study, we used aortic samples collected during autopsies performed within 4–6 hrs after accidental death on 12 men and 4 women aged 31–57 years. The vessels were dissected longitudinally and were washed with phosphate-buffered saline (PBS). Upon macroscopic examination, aortic areas, relevant to the aims of the study, were cut-off and each of the dissected fragments was divided into parts, for biochemical and histological/morphological analyses. Based on both macroscopic and histological examinations, tissue specimens were classified either as tissue samples from the normal intima or as tissue samples from the early atherosclerotically injured areas, including Types I and Type II lesions, *i.e*. initial lesions and fatty streaks (according to the American Heart Association classification 2, 3). The available characteristics of the donors and post-mortem intervals are summarized in Table 1. For further mRNA measurements, tissue samples were frozen in liquid nitrogen and kept at −70°C. To compare the expression of the selected genes between the normal aorta and atherosclerotic lesions, mRNA measurements were performed in pairs of ‘intact tissue fragment/atherosclerotically injured fragment’; tissue fragments for each pair were taken from the same donor. For a comparison between the normal aorta and type I lesions, 12 pairs of tissue fragments were analysed whereas for a comparison between the normal aorta and type II lesions, 15 pairs of tissue fragments were analysed.

**Table 1:** Characteristics of autopsy material

Case #	Sex	Age (years)	PMI (hours)	Types of lesion identified*^,^†	Types of lesion studied *^,^†
1	M	31	4.5	I, II	I, II
2	M	31	4	I, II, Vc‡	I, II
3	M	39	5	I, II	I, II
4	M	40	6	I, II, Vc	I, II
5	M	46	5.5	II, Va	II
6	M	48	5	II	II
7	M	50	4	I, II, Va	II
8	M	51	4	I, Va, Vc	I
9	M	51	6	II	II
10	M	52	4.5	I, II	I, II
11	M	53	4.5	I, II, Va	I, II
12	M	55	4	I, II, Va, Vc	I, II
13	F	39	4	I, II, Va	I, II
14	F	44	5.5	I, II	I, II
15	F	54	4	I, II	I, II
16	F	57	4.5	I, II	I, II

M: Male; F: Female; PMI: Post-mortem intervals.

* All aortas contained areas with the normal (undiseased) intima (not shown in the table).

† AHA classification 2, 3.

‡ Type V lesions are defined as lesions in which prominent new fibrous connective tissue has formed 2, 3. When the new tissue is part of a lesion with a lipid core (type IV), this type of morphology may be referred to as fibroatheroma or type Va lesion. A type V lesion in which the lipid core and other parts of the lesion are calcified may be referred to as type Vb. A type V lesion in which a lipid core is absent and lipid in general is minimal may be referred to as type Vc. 2, 3.

### Routine electron microscopy

For routine electron microscopic analysis, tissue samples (∼1 mm^3^ each) were cut in 1% glutaraldehyde in 0.1 M sodium cacodylate buffer (pH 7.4), fixed in the same solution and routinely processed and embedded in Araldite resin as used previously 23. Ultrathin sections taken from each Araldite block were stained with uranyl acetate and lead citrate and examined with the aid of a Hitachi H7000. The identification of cell types in ultrathin sections was carried out as detailed in our earlier publication 24.

### Electron microscopic immunohistochemistry

Subcellular localization of CD68 antigen was studied in ultrathin cryosections. For this, tissue samples were fixed in 2% formaldehyde and 0.2% glutaraldehyde in 0.1 M PBS, pH 7.4, and prepared for cryosectioning and immunogold labelling as described by Slot *et al*. 25 with the slight modification proposed by Liou *et al*. 26 in which sections were picked up in a 1:1 mixture of methyl cellulose and 2.3 M sucrose, resulting in an improved ultrastructure 26. After blocking the sections by floating the grids on drops of blocking buffer [1% bovine serum albumin (BSA), 0.02 M glycine, 10% cold water fish gelatine in PBS] for at least 30 min., the primary anti-CD68 antibody (Dako, CD68; EBM11; Gbostrup, Denmark) was applied at a 1:50 dilution in blocking buffer. After incubating for 1 hr at 37°C or over night at 4°C in a humid chamber, excess antibody was removed by washing six times (3 min. each time) in drops of PBS. Secondary antibody conjugated with gold particles was applied for 30 min.–1 hr at 37°C diluted in blocking buffer. The labelled grids were washed six times in PBS and five times in distilled water prior to negative contrasting and embedding in methyl cellulose. For this, the sections were floated on three consecutive drops of 0.2% uranyl acetate and 2% methyl cellulose in distilled water for 2.5 min. each before blotting off excess contrasting solution and air drying the grids. All reagents used in the above procedures were obtained from British Biocell International (UK). In addition, the localization of CD68 was examined in Lowicryl-embedded tissue specimens according to the procedures detailed elsewhere 27. In all experiments, appropriate controls were performed which resulted in the absence of immunopositivity. The distribution and localization of CD68 antigen were analysed with the aid of a Hitachi H7000.

### Selection of markers important in the functioning of the vacuolar-lysosomal system

As markers that are important in the functioning of the vacuolar-lysosomal system we chose the following markers: the early endosome antigen 1 (EEA1) and interacting with EEA1 – a small GTPase, Rab5a (responsible for translocation and fusion of early endosomes 28, 29), lysosome-associated membrane glycoproteins 1 and 2 (Lamp1 and Lamp2) (responsible for biogenesis of lysosomes, translocation and fusion of phagosomes with lysosomes 30), protein p62 (responsible for delivery of ubiquitin-tagged proteins into autophagosomes 31) and antigen CD63 (responsible for exocytosis and possibly for biogenesis of lysosomes 32). Lamp1, Lamp2, p62 and CD63 can be displaced between the plasma membrane, late endosomes and lysosomes and thus, their levels characterize the entire vacuolar-lysosomal system 30–32. We also investigated changes and lysosomal distribution of CD68 antigen. Even though, in some conditions, CD68 antigen can be expressed by various hematopoietic and non-hematopoietic cell types 33–35, it is well known that CD68 is predominately expressed by macrophages and thus, is often used as an identification marker for macrophagal cells 33–36. CD68 represents a glycoprotein of the LAMP family which binds to low-density lipoproteins (LDL) and plays important roles in phagocytotic activity and in intracellular lysosomal metabolism 33–35.

### RNA isolation and real-time polymerase chain reaction (PCR)

RNA was isolated by a homogenization of the frozen samples taken from the vessel walls in TRIzol Reagent (Invitrogen; Carlsbad, CA, USA). The RNA was then treated with chloroform, precipitated with isopropanol and washed with ethanol. The synthesis of cDNA was performed on 2–6 μg of total RNA using a Promega ImProm_II™ Reverse Transcription System kit (Promega Corporation; Madison, WI, USA). The synthesized cDNA was used as a template for quantitative real-time polymerase chain reaction (qRT-PCR) on a Rotor-Gene 3000 amplifier (Corbett Research, Sydney, Australia) with a kit of reagents including the intercalating dye SYBR Green I (Syntol, Moscow, Russia) as recommended by the manufacturer. The details of amplification were described earlier 37. The primers for the most part of mRNAs were chosen using the Beacon Designer 6.00 program (http://www.PremierBiosoft.com) and described previously 38. The primers for GNB2L1 were the same as described by Ishii *et al*. 39. The primers for analysis of endosomes (phagosomes) and lysosomes are provided in Table 2. To check for the absence of products amplified from genomic DNA, isolated RNA was used as a template. Amplified products were sequenced using an ABI PRISM® BigDye™ Terminator v.3.1 kit of reagents and an ABI PRISM 3100-Avant automated DNA sequencer to confirm the expected sequence. The results were included only when the melting temperature and the electrophoretic mobility of the amplified products corresponded to the expected values. DNA sequencing was performed in the Genome Interinstitutional Collective Use Center of the Engelhardt Institute (http://www.genome_centre.narod.ru/), supported by the Russian Foundation for Basic Research (project no. 00-04-55000). The content of the particular mRNAs was normalized to glyceraldehyde-3-phosphate dehydrogenase (GAPDH) mRNA content as an internal reference control and expressed as percent.

**Table 2:** Primers used for measurements of mRNAs relevant to the endosomal/lysosomal compartment

mRNA	Sequence	Size of PCR product, bp
EEA1	For 5′-GGAGGAGAGTCTAATCTTGCTTTG-3′	182
	Rev 5′-GAATCAGTCACCAACCCATCAG-3′	
Rab5a	For 5′-CAGTTCAAACTAGTACTTCTGG-3′	200
	Rev 5′-GCTAGGCTATGGTATCGTTCTTG-3′	
Lamp1	For 5′-AACTTCTCTGCTGCCTTCTC-3′	172
	Rev 5′-GAGTGAGTGTATGTCCTCTTCC-3′	
Lamp2	For 5′-GATACTTGTCTGCTGGCTACC-3′	222
	Rev 5′-CATGCTGATGTTCACTTCCTTC-3	
p62 lck	For 5′-CCGAGTGTGAATTTCCTGAAG-3′	144
	Rev 5′-CTCTGTGCTGGAACTCTCTG-3′	
CD63	For 5′-GTGTGAAGTTCTTGCTCTACG-3′	154
	Rev 5′-ACTGCGATGATGACCACTG-3′	
CD68	For 5′-ATTCATGCAGGACCTCCAGC-3′	263
	Rev 5′-AGGAGAAACTTTGCCCAAAG-3′	
GAPDH	For 5′-GAGCCCGCAGCCTCCCGCT-3′	145
	Rev 5′-GCGCCCAATACGACCAAATC-3′	
GNB2L1	For 5′-GAGTGTGGCCTTCTCCTCTG-3′	224
	Rev 5′-GCTTGCAGTTAGCCAGGTTC-3′	

### Statistical analysis

The results were analysed with the Statistica 7.0 program. Independent samples were compared using the Mann–Whitney U-test. The data were analysed on normality by distribution fitting. Both mRNA contents in samples and ratios of mRNA contents in paired samples (lesion/intact tissue) did not correspond to normal distribution. Therefore, nonparametric criteria for analysis of changes in mRNA contents and correlations between mRNAs were used. The contents of mRNA in intact aorta and lesions of types I and II did not show gender differences or age dependence and thus, combined data for samples from male and female aortas excised from donors of different ages were used. Correlations were evaluated using the Spearman rank test. Differences or correlations were considered significant at *P* < 0.05. In this report, mean ± SD values are shown.

## Results

### Ultrastructural observations

**Fig. 1:**
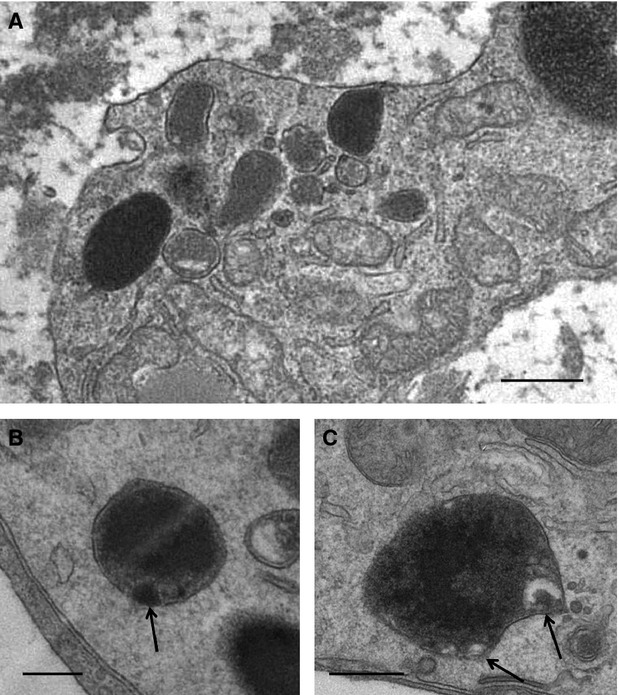
Structural appearance of lysosomes in the normal intima (**A**) and in the initial atherosclerotic lesions (Type I lesion) (**B** and **C**) in the human aorta. In (**A**), note that lysosomes are round- and oval-shaped structures characterized by the presence of homogenous material of middle or high electron density. In (**B** and **C**), inclusions within lysosomes are shown by arrows. (**A**–**C**): Electron microscopy. Scale bars = 500 nm (**A**), 200 nm (**B** and **C**).

**Fig. 2:**
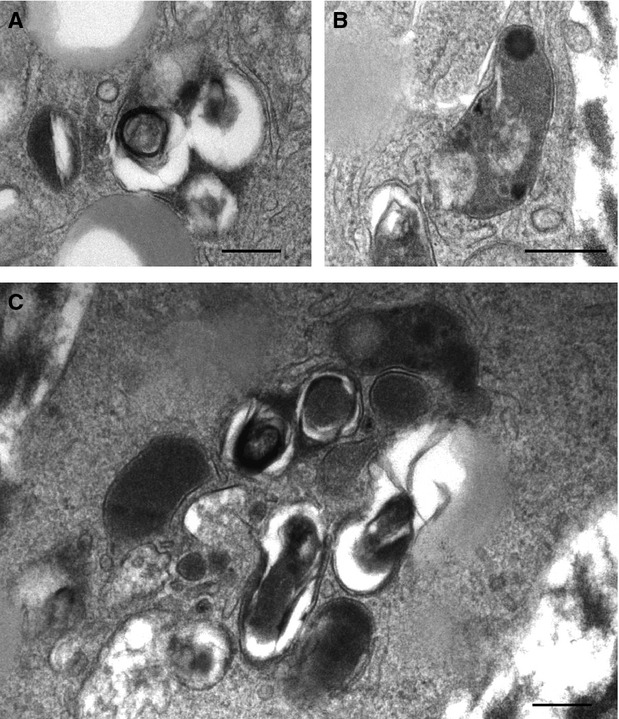
Structural appearance of lysosomes in intimal cells containing ‘lipid droplets’ in intimal cells in fatty streaks (Type II lesions; **A**–**C**). Note that, while few lysosomes are characterized by the presence of homogenous material of middle or high electron density, the majority of lysosomes are represented by secondary lysosomes and autophagosomes, containing lipid inclusions. (A–C): Electron microscopy. Scale bars = 200 nm (A–C).

Ultrastructural examination of numerous profiles of cells residing in the normal intima revealed characteristic round and oval shapes of lysosomes (Fig. 1A). In these cells, lysosomes were filled with a homogenous material of middle or high electron density (Fig. 1A). In the initial lesions (Type I lesion), the presence of inclusions within some lysosomes were detected (Fig. 1B and C). Electron density of the lysosomal ‘matrix’ was quite irregular in these lysosomes (Fig. 1B and C). The cells, containing some lipid inclusions in the initial lesions contained a well-developed vacuolar-lysosomal apparatus while they did not contain visible filaments or the basal membrane and thus were identified as macrophages. In cells in fatty streaks (Type II lesion), some lysosomes were presented by secondary lysosomes and autophagosomes which contained lipid inclusions (Fig. 2A–C). Some of the lipid-laden cells in fatty streaks contained a large number of lipid inclusions and autophagosomes.

**Fig. 3:**
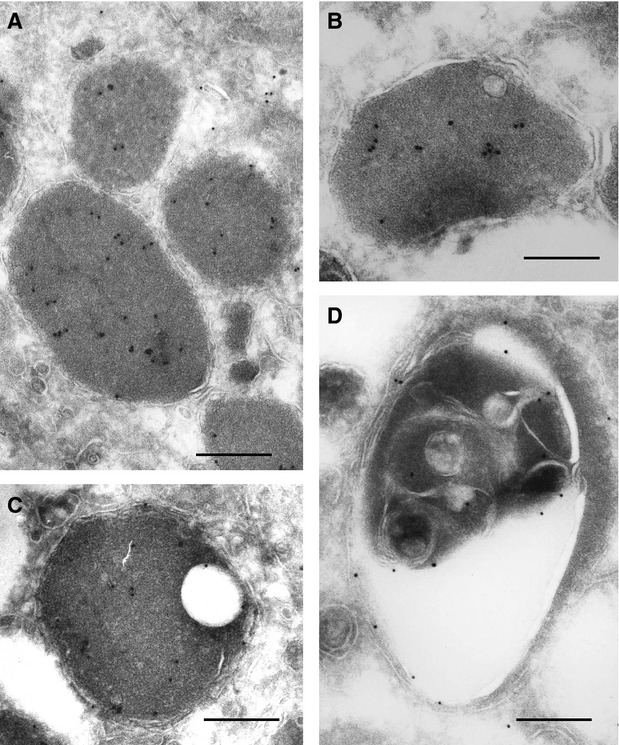
Electron microscopic immunocytochemical demonstration of the distribution of CD68 antigen in lysosomes in cells located in the normal intima (**A**), the initial lesions (Type I lesions; **B** and **C**) and a fatty streak (**D**) of the human aorta. (A–D): Electron microscopy; Immunogold technique. Scale bars = 200 nm (A–C).

**Fig. 4:**
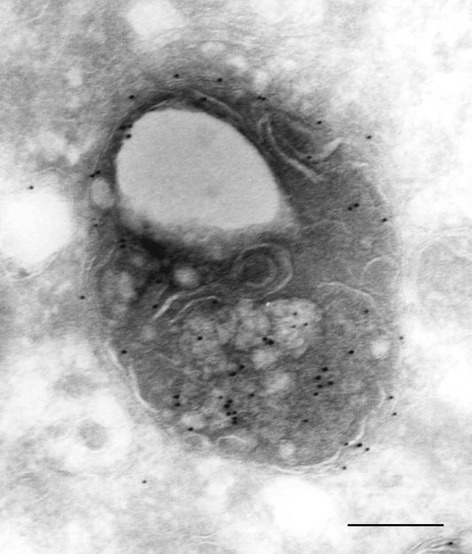
A high resolution micrograph showing the distribution of CD68 antigen in an autophagosome in an intimal cell in a fatty streak of the human aorta. Electron microscopic immunocytochemistry; Immunogold technique. Scale bar = 200 nm.

Examination of cryosections at the electron microscopic level revealed the same ultrastructural organization (Fig. 3A–D) that was observed by routine electron microscopy except that there was also a pronounced presence of lamellar bodies in cells residing in fatty streaks (Type II lesion; Figs 3D and 4). These lamellar bodies (or ‘myelin-like figures’) consisted of membrane-bounded cytoplasmic ‘vesicles’ containing a series of concentric, membrane-like swirls (Figs 3D and 4). The membranes that surrounded the lamellar bodies frequently were found to be discontinuous or disrupted (Figs 3D and 4). Lamellar bodies, surrounded by membranes, also frequently contained an amorphous electron-dense substance (Figs 3D and 4).

Immunocytochemical immunogold staining of cryosections with anti-CD68 revealed a specific association of CD68 antigen with primary and secondary lysosomes as well as with autophagosomes (Figs 3A–D and 4). It has been found that, while in primary lysosomes the distribution of immunogold-labelled CD68 antigen was quite regular throughout the lysosomal ‘bodies’ (Fig. 3A), in the secondary lysosomes and, especially, in autophagosomes, CD68 antigen was distributed irregularly (Figs 3D and 4).

### Data obtained by PCR analysis

To validate the relevancy of GAPDH mRNA as a reference point, we first analysed the expression of an additional housekeeping gene GNB2L1, in both intact and atherosclerotically injured aortic fragments obtained from several donors. The content ratios of GNB2L1 mRNA in atherosclerotically injured areas to that in the intact aorta fragments (normalized by GAPDH mRNA), were close to 1.0 for atherosclerotically injured areas (Type I and Type II lesions) [0.94 ± 0.38 (*n* = 6) and 0.96 ± 0.24 (*n* = 6) respectively]. This suggested that the expression of the two housekeeping genes does not vary significantly during early atherogenesis.

**Fig. 5:**
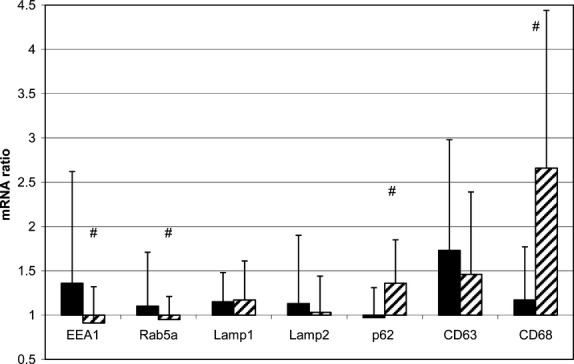
The ratios of the contents of mRNAs encoding for the components of endosome/lysosome compartment in pairs of atherosclerotically injured/intact human aorta. Black bars: initial lesion/intact tissue ratios; hatched bars: fatty streak/intact tissue ratios. Symbol # shows significant difference from 1.0.

As seen from Figure 5, no significant changes in mRNAs that encode the components of endosome/lysosome compartment were found in the initial lesion (Type I lesion) of the aorta. However, in fatty streaks (Type II lesion), the contents of EEA1 and Rab5a mRNAs diminished while the contents of p62 and CD68 mRNAs were increased compared with the intact tissue. Prominent changes were observed in coupling between the contents of mRNAs encoding for molecules that govern the functioning of endosomes and lysosomes in both Type I and Type II lesions. Although the total amounts of correlations were comparable in the intact and atherosclerotically injured areas tissues (7, 9 and 6 in intact tissue, initial lesion and fatty streak respectively), the distribution of correlations between mRNAs varied in the different types of atherosclerotic lesions.

When the correlations in three analysed tissue types (the normal aortic areas, Type I lesion and Type II lesion) were superposed, a number of these correlations appeared to be conservative, *i.e*. were common for, at least, two tissues (Table 3). The distribution of these conservative ties was not uniform in three analysed tissue types; in the initial lesions (Type I lesion) and fatty streaks (Type II lesion), the proportions of such correlations were significantly lower (56% and 50% respectively) than in the intact tissue (86%). The most conservative ties included: EEA1-Rab5a and Lamp2-CD68.

**Table 3:** Correlations between the contents of mRNAs encoding for components of endosome/lysosome compartment in normal (undiseased) and atherosclerotically injured areas of the human aorta. Positive correlations are shown as black boxes. Single and double symbols • mark correlations common for two and three tissue types respectively

mRNA species	Intact tissue						Initial lesion						Fatty streak					
		
	Rab5a	Lamp1	Lamp2	p62	CD63	CD68	Rab5a	Lamp1	Lamp2	p62	CD63	CD68	Rab5a	Lamp1	Lamp2	p62	CD63	CD68
EEA1	••	•					••						••	•				
Rab5a		•	•					•	•									
Lamp1			•						•									
Lamp2						••						••						••
p62																		
CD63																		

## Discussion

This study, for the first time, investigated parameters of the regulatory system of expression of genes relating to the lysosomal function of intimal cells residing in the initial lesion (Type I Lesion).

A comparative analysis of the correlations between mRNAs in the regulatory system of expression of lysosome-relevant genes in the ‘normal intima-initial lesion-fatty streak’ sequence revealed a certain rearrangement. In particular, it has been found that the expression of CD68 antigen is dramatically increased in fatty streak, compared with the initial lesion type. This is not surprising as it is well known that the penetration of monocytes from the blood stream through the endothelial barrier into the subendothelial layer and the following differentiation of the majority of monocytes into CD68^(+)^ macrophages represent the earliest events in the development of the disease 1, 15, 18, 40.

Apart from the demonstration of the increase in CD68 mRNA in the earliest stages of atherogenesis, electron microscopic immunocytochemistry revealed that there were certain changes in the distribution of CD68 antigen in lysosomes along the ‘normal intima-initial lesion-fatty streak’ sequence. Specifically, the study found that, while the distribution of CD68 antigen is quite regular throughout the lysosomal ‘bodies’ of intimacytes that reside in the normal arterial wall, CD68 antigen becomes distributed in irregular patterns in lysosomes in intimacytes in developing atherosclerotic lesions. In parallel with the altered patterns of CD68 distribution, the notable changes in the structural appearance of lysosomes in the ‘normal intima-initial lesion-fatty streak’ sequence were observed as well. These changes included the alteration in electron density of the ‘matrix’ of lysosomal ‘bodies’ as well as the accumulation of non-catabolized lipid inclusions within the lysosomes. The use of cryo-preserved tissue specimens allowed us to identify the presence of lamellar bodies within lysosomes in cells residing in fatty streaks.

These lysosomal lamellar bodies which can be also described as ‘myelin-like figures’ were found to consist of membrane-bounded cytoplasmic ‘vesicles’ containing a series of concentric, membrane-like swirls. Frequently, the membranes that surrounded the lamellar bodies were found to be discontinuous or disrupted. Similar lamellar bodies have been observed by others in lysosomes in a number of conditions related to pathological lysosomal stress 41. In Tay-Sachs disease, a hereditary disorder of glycolipid metabolism with massive cerebral accumulation of GM2 gangliosides, which results from the absence of lysosomal enzyme 3-D-*N*-acetylhexosaminidase, the abundance of lamellar bodies has been reported 42. Also, in an experimental study, the induction of intracellular ganglioside storage by ‘feeding’ either gangliosides or sulfatides to cultured spinal cord or dorsal root ganglia was found to give rise to lamellar body formation 43. Lamellar bodies were also observed in I-cell disease which involves deficiencies in several lysosomal enzymes because of an impaired phosphorylation of the enzymes that leads to a failure to re-accumulate the excreted enzymes 44. The functional significance of the formation of lamellar bodies in lysosomes in various pathologies is not yet understood, but there is a view that the formation of lamellar bodies might reflect not gene defects but rather reflects the existence of ‘the common crossroad of a variety of altered metabolic pathways and therefore do not, by themselves, point to a specific defect’ 41. In any case, the fact that lysosomal lamellar bodies are absent in the normal intima of the aortic wall, but appear in cells in fatty streaks might indicate that their formation is relevant to the pathogenetic mechanisms which become involved in the development of atherosclerosis.

In this study, apart from the analysis of CD68 antigen, we examined also other key markers of endosome/lysosome compartments, including EEA1, Rab5a, Lamp1, Lamp2, protein p62 and antigen CD63. Interestingly, this study showed no significant changes in the analysed mRNAs encoding for the components of endosome/lysosome compartment in the initial lesions (Type I lesion) of the aorta, compared with those in the normal intima, but in fatty streaks (Type II lesion), the contents of EEA1 and Rab5a mRNAs were found to be diminished while the contents of p62 and CD68 mRNAs were increased, compared with the intact tissue. There were prominent changes in coupling between the contents of the analysed mRNAs encoding for molecules that govern the functioning of endosomes and lysosomes in both Type I and Type II lesions. Although the total amounts of correlations were comparable in the normal and atherosclerotically injured tissues (7, 9 and 6 in ‘intact’ tissue, initial lesion and fatty streak respectively), the distribution of correlations between mRNAs was found to vary in the different types of lesions. When the correlations in the three analysed tissue types (the normal aortic areas, Type I lesion and Type II lesion) were superposed, a number of these correlations appeared to be conservative, *i.e*. were common for at least two tissues. The distribution of these conservative ties was not uniform in the three analysed tissue types; in the initial lesion (Type I lesion) and fatty streak (Type II lesion), the proportion of such correlations was significantly lower (56% and 50% respectively) than in the intact tissue (86%). The most conservative ties were found in EEA1-Rab5a and Lamp2-CD68 pairs. Although the interpretation of this kind of data can always be open to criticism, one can suggest that the coordinated expression of EEA1 and Rab5a at the level of transcription provides a mechanistic base for their functioning in tight cooperation. CD68 is known to be an integral component of the intracellular transport that involves lysosomes which are largely built up of Lamp1 and Lamp2 30–35. This is in agreement with the results of our study that demonstrated a high conservatism of correlations between CD68 and Lamp2 mRNAs. The conservation of correlations between Lamp2 and CD68 mRNAs, found for the first time in the present work, might reflect the adaptation of the lysosomal compartment to the entrance of lipids *via* scavenger receptors. High conservatism of correlations between mRNA contents in EEA1-Rab5a and Lamp2-CD68 pairs can indicate the existence of common regulatory elements in the respective genes, with the elements being functional and dominant in both intact intima and early lesions of types I and II. This prediction can be helpful for future structure functional analysis of regulatory gene regions. In this aspect, less conservative ties should be also taken into account.

It is essential to note here that in this study, we measured the mRNAs in a mixture of different cell types and phenotypically different cells. The identified correlations likely reflect the coordination of transcriptional activity of the corresponding pairs of genes in the predominant cell population(s) intima; in the normal intima the vast majority of cells are well known to be smooth muscle cells while in fatty streaks, apart from smooth muscle cells, macrophages constitute a notable portion of the total number of intimal cells 1–3, 45. The high correlation conservatism of Lamp2-CD68 pair in the ‘normal intima-initial lesion-fatty streak’ sequence might reflect the consistency of these genes in different cell populations (CD68 is expressed predominantly in macrophages 34, 35, but macrophages in the intact aortic tissue are quite rare cells 2, 3, 45).

In the 1980s of the last century, a group of mathematicians 46, 47 put forward a concept of ‘correlation adaptometry’, according to which adverse conditions (diseases, climatic factors, *etc*., at various levels of their natural organization, from a molecular level up to the biocenosis level) induce an increase in the degree of entanglement between different parameters of biological systems. According to this concept, the entanglement between different parameters is expressed by the number and strength of correlations between parameters 46. The application of this concept to the results obtained in this study might lead to a suggestion that the initial lesion (Type I lesion) is not a stage of the pathological process, but it is rather a stage of the ‘adaptation’ (absence of significant rise in the total number of correlations between the mRNA, compared with the norm along with decrease in the proportion of conservative correlations). In this stage, the vast majority of intimal cells become involved in contact with the ‘dangerous signals’, such as LDL excess and modified LDL that appear to be present in the intimal extracellular space in early atherogenesis 15. Thus, according to the above concept, the initial lesion might also be described as a pre-disease stage rather than a disease stage. One can consider that the pre-disease stage can be regarded also as the stage of ‘unstable’ adaptation 48. According to the concept of ‘correlation adaptometry’ 46, 47, one would expect that the pathologic processes that lead to the formation of fatty streak would be reflected in an increase in the degree of the conjugacy of the expressions of the analysed genes. However, this was not observed in the present study, suggesting the occurrence of the dysregulation in the expression of the lysosome-relating genes. One can speculate that this dysregulation may contribute to the inability of lysosomes to cope with a high intake of lipids in developing fatty streaks. The clinical relevance of the above speculations is that the pre-disease stage might be spontaneously reversible, provided that the harmful factors are removed 48, 49 while the removal of the disease-initiating factors at the stage of disease (such as fatty streak, in this particular case) might not be accompanied by the disappearance of the lesion without therapeutic intervention 2, 3.

In general, the findings of this study do not contradict the hypothesis of de Duve that postulates that lysosomes in developing atherosclerotic lesions acquire ‘functional deficiency’ or are ‘initially’ characterized by ‘inferiority’ of lysosomes in sites of developing lesions 11–14. Although one can argue that the molecular content of the extracellular microenvironment (in particular, the formation of modified lipoproteins) in developing atherosclerotic lesions changes from that of the normal intima and that ‘intact’ lysosomes simply cannot catabolize modified lipoproteins and other modified molecules, it is worth noting here that some modified lipoproteins circulate in the blood and likely penetrate other tissues which contain macrophages, but foam cell formation is largely restricted to the arterial wall 1, 15, 17, 18. Obviously, the mechanisms which are responsible for the occurrence of the peculiarities of lysosomes in early atherogenesis require further investigation.

Structural abnormalities in both lysosomes and lysosome-related organelles have been observed in a number of human genetic diseases such as the Chediak-Higashi and Hermansky-Pudlak syndromes 17, 19, 21, 50, 51. In contrast to classic lysosomal storage diseases, in which mutations in specific genes occur (for example, in the LYST gene in Chédiak–Higashi syndrome), there is no evidence that in atherosclerosis a specific gene could have a defect. This study indicates that the expression of a group of genes is altered and this might explain the observed structural alterations of lysosomes. Importantly, the structural alterations of lysosomes revealed in this study are similar with those described in classic lysosomal storage diseases, including the accumulation of unmetabolized lipids 17, 19, 21, 50, 51. In classic lysosomal storage diseases, the functional deficiency of structurally altered lysosomes is demonstrated 17, 21, 50. As in early atherosclerosis, the structural alterations of lysosomes were found to resemble those of classic lysosome-relating gene disorders, it is reasonable to suggest that the alterations of lysosomes, identified in this study, might have an important impact on the functioning of intimal cells and thus on the development of atherosclerosis.
